# Characterization of Guanine Deaminase from *Kluyveromyces marxianus* and Its Industrial Application to Reduce Guanine Content in Beer

**DOI:** 10.3390/foods14071085

**Published:** 2025-03-21

**Authors:** Peng Zhou, Junhao Xu, Zixuan Wang, Baoguo Li, Zhijun Zhao

**Affiliations:** 1 School of Health Science and Engineering, University of Shanghai for Science and Technology, Shanghai 200120, China; 211310154@st.usst.edu.cn (P.Z.); 223392705@st.usst.edu.cn (Z.W.); 2 Laboratory of Biorefinery, Shanghai Advanced Research Institute, Chinese Academy of Sciences, Shanghai 200120, China; 3 College of Life Science, Northeast Forestry University, Harbin 150040, China; xujunhao@nefu.edu.cn

**Keywords:** guanine deaminase, *Kluyveromyces marxianus*, purine, beer

## Abstract

Guanine deaminase (GDA) catalyzes the first step in purine catabolism by converting guanine to xanthine. Despite its significant role in the development of low-purine food, studies on GDA remain limited compared to other metabolic deaminases. To identify a GDA with high enzyme activity and appropriate optimum parameters, GDAs from *Kluyveromyces lactis*, *Kluyveromyces marxianus*, *Lentilactobacillus kefiri*, and *Lactobacillus buchneri* were heterologously expressed in *Escherichia coli*. The GDA from *Kluyveromyces marxianus* (KM-GD) showed the most potent enzyme activity (2.21 IU/mL) at 30 °C and pH 6.5, which is close to the pH of saccharified wort. Furthermore, analyzing the crystal structures of GDAs from different sources revealed that hydrogen bonds could enhance substrate affinity and strengthen enzyme activity. In addition, active pockets with an appropriate size may contribute to high enzyme activity. Finally, KM-GD helped reduce guanine by 80.33% in beer wort and by 80.00% in matured beer, thus suggesting its promise for industrial application in low-purine food production.

## 1. Introduction

Guanine deaminase (EC 3.5.4.3, GDA) belongs to the amidohydrolase superfamily (AHS). The AHS is one of the most functionally important and diverse protein superfamilies, with more than 100,000 members identified. AHS members all share a highly conserved (β/α)_8_ TIM barrel fold and typically utilize a divalent metal center to mediate the hydrolysis of amide or ester bond-containing substrates [1,2]. GDA plays an important role in the purine degradation pathway. It catalyzes the first step in purine catabolism by converting guanine and water to xanthine and ammonia via hydrolytic deamination [3,4]. GDA is involved in a wide range of life activities. Studies have shown that it impacts neuronal morphology, and it has been implicated in traumatic brain injury, memory dysfunction, and psychiatric diseases [5,6,7,8]. In addition, GDA is associated with cellular microtubule formation [9] and melasma lesions [10]. GDA leads to the downregulation of melanin levels, suggesting its role in skin hyper-pigmentary disorders [11].

GDA was first described in 1932 [12]; since then, many GDA sources have been studied and reported. It is found in bacteria, lower eukaryotes, plants, and higher eukaryotes [13]. To date, purine-degrading enzymes from *Arxula adeninivorans* [14], *Kluyveromyces lactis* [15], and *Alkalihalobacillus clausii* [16] have been characterized and applied in low-purine food production. The purine content in beef stock and rolled fillet of ham was reduced successfully with an enzyme mixture containing guanine deaminase from *Arxula adeninivorans*. Adenine deaminase and guanine deaminase from *Kluyveromyces lactis*, in combination with other purine-degrading enzymes, achieved marked reductions in the purine content of beer samples. These results indicate the high feasibility of using enzymes to produce low-purine foods.

Beer is a low-alcohol beverage rich in carbon dioxide; it is brewed using yeast and fermented with barley malt, hops, and water as the main raw materials [17]. Beer contains guanosine, guanine, inosine, hypoxanthine, xanthine, adenine, adenosine, and other purine substances, and it has the highest purine content among common alcoholic beverages. An excessive intake of beer tends to cause the accumulation of uric acid, which induces gout [18,19]. Most of the purines in beer are derived from malt and mainly consist of nucleotides and bases [20,21,22]. It is worth noting that the purine content in beer is as high as 60~100 mg/L, and it can reach more than 100 mg/L in some whole-wheat beer [23]. Most studies have found significant differences in the purine content of different beer samples, with all emphasizing that guanosine is the most abundant purine, accounting for 40 percent of the total purine content [24,25,26]. With the consumption of beer rising year by year, the number of patients with hyperuricemia and gout is also increasing year by year worldwide. Reducing guanine levels is an important step in reducing the total purines in beer, and it can lower the risk of hyperuricemia and gout caused by drinking beer.

In this study, four different sources of GDA were heterologously expressed in *E. coli* and their enzyme activities were compared. The enzymatic properties of GDA from *Kluyveromyces marxianus* (KM-GD) were evaluated. Moreover, active pockets and critical amino acid residues of guanine obtained from *Kluyveromyces marxianus*, *Kluyveromyces lactis*, and *Lactobacillus buchneri* were identified and compared. Three critical residues (ASP307, LYS358, and ASN417) in KM-GD that collectively mediate hydrogen bonding interactions with the guanine substrate were considered, forming a total of three stabilizing contacts. We propose this synergistic interaction mechanism enhances catalytic efficiency through two complementary effects: steric complementarity—an optimally sized active pocket facilitates unhindered substrate access; and electrostatic preorganization—coordinated hydrogen bonds lower activation energy by pre-aligning the substrate for nucleophilic attack. Notably, structural limitations in other GDAs were identified: KL-GD and LB-GD possess recessed active pockets with constricted apertures. These geometric constraints create a kinetic barrier. Finally, KM-GD helped produce low-purine beer, significantly decreasing the guanine content.

## 2. Materials and Methods

### 2.1. General Bacterial Strains, Plasmids, Media, and Culture Conditions

*Escherichia coli* JM109 was used for plasmid replication, and *E. coli* BL21 (DE3) was utilized for guanine deaminase gene expression. Plasmid pCDFDuet-1 was obtained from Novagen (Darmstadt, Germany). Cultures were performed in Luria–Bertani seed medium for plasmid replication and in Terrific Broth medium for expression (a: yeast extract 23.6 g/L, tryptone 11.8 g/L, sterilized at 121 °C for 20 min; b: 5 mL of 80% glycerol, 100 mL phosphate solution; 94 g/L K_2_HPO_4_, 22 g/L KH_2_PO_4_, sterilized at 115 °C for 20 min, and combined) [16].

### 2.2. Nucleic Acid Manipulations

The full-length amino acid sequences of *Kluyveromyces lactis*, *Kluyveromyces marxianus*, *Lentilactobacillus kefiri*, and *Lactobacillus buchneri* were procured from the National Center for Biotechnology Information Database (NCBI). Whole-gene synthesis was conducted by Sangong Bioengineering (Shanghai, China).

### 2.3. Overexpression and Purification of Guanine Deaminase from Different Sources

The engineered bacterial strains were incubated inside a shake-flask LB culture overnight at 37 °C and 200 rpm. Then, the strains were transferred to fresh TB fermentation medium at OD_600_ 0.1 and incubated at 37 °C and 200 rpm to OD_600_ 1.75. The bacteria were induced with 1 mmol/L IPTG (isopropyl-β-D-thiogalactopyranoside) for 20 h at 20 °C and 200 rpm.

The induced *E. coli* cells were collected via centrifugation for 10 min at 8000× *g* and 4 °C. After washing with 10 mM phosphate buffer (pH 7.4), the cells were resuspended in 15 mL of a cell disruption buffer (20 mM Tris, 500 mM NaCl, 1% triton-X100, 1 mM β-mercaptoethanol, pH 7.4). The cell suspension was disrupted via an ultrasonic processor: XO-400SD (Nanjing Xianou Instruments Manufacture Co., Ltd., Nanjing, China). Cell debris was removed via centrifugation (9000× *g* for 10 min at 4 °C), and the supernatant was designated as the crude enzyme. After being filtered through a 0.22 μm pore size membrane (Millipore, Boston, MA, USA), the recombinant enzymes were purified by affinity chromatography on nickel Ni-NTA resin. After washing with 20 mM Tris (pH 7.4) containing 20 mM imidazole in turn, the recombinant proteins were eluted with 20 mM Tris (pH 7.5) containing 500 mM imidazole. The purified proteins were determined via 10% sodium dodecyl sulfate–polyacrylamide gel electrophoresis (SDS-PAGE), and the protein concentrations were measured using the Bradford method, with bovine serum albumin as the standard.

### 2.4. Guanine Deaminase Enzyme Activity

The guanine deaminase activities of *Kluyveromyces lactis* (KL-GD), *Kluyveromyces marxianus* (KM-GD), *Lentilactobacillus kefiri* (LK-GD), and *Lactobacillus buchneri* (LB-GD) were measured via the generation of xanthine at 293 nm. Each 2 mL reaction was composed of 200 μL of 20 mmol/L guanine and 1.8 mL of 50 mmol/L KH_2_PO_4_-K_2_HPO_4_. Then, 20 μL of enzyme solution was added and placed at 37 °C for 10 min. The reaction was stopped by adding 200 μL of 20% KOH to inactivate the enzyme [15].

The effect of pH on guanine deaminase activity was examined in buffers ranging from pH 3.0 to 10.0. The buffer solutions included 50 mM sodium acetate (pH 3.0–5.0), 50 mM sodium phosphate (pH 5.5–8.0), and 50 mM Tris-HC1 (pH 8.5–10.0). The effect of temperature on guanine deaminase activity was investigated at temperatures ranging from 20 to 60 °C. Thermostability was determined by measuring the residual activity after incubating guanine deaminase at temperatures between 20 and 60 °C for 30 min. pH-dependent stability was assayed by determining the residual activities after incubating the enzymes in 50 mM buffers (pH 3.0–10.0) for 24 h at room temperature (25 °C) [27].

### 2.5. Homology Modeling and Active Pocket Prediction

A homodimer 3D structure of guanine deaminase was constructed with the Swiss model (http://www.swissmodel.expasy.org, accessed on 12 December 2024) using the amino acid sequence produced by the EXPASY Translate tool (https://web.expasy.org/translate/, accessed on 12 December 2024). The monomer structures of KL-GD, KM-GD, and LB-GD were constructed using I-TASSER. The default parameters were utilized for each molecular model. PyMOL software was used for protein dehydration and ligand removal. AutoDock Tools 1.5.6 was used to hydrogenate receptor proteins and store them in PDBQT format. AutoDock was used to dock the guanine based on its affinity. Sorting and force scoring were used to determine the conformation with the lowest binding free energy. PyMOL 2.5.4 was used for image analysis and to visualize the results. The active pocket was predicted using DoGSiteScorer (https://proteins.plus/, accessed on 23 December 2024).

### 2.6. Guanine Degradation by KM-GD in Beer

Malted barley (crushed to 0.5–1.0 mm particle size) and brewing water (adjusted to pH 5.2–5.5 with lactic acid) were used as raw materials. Coarsely ground malt (2.5 kg) was mixed with water at a ratio of 1:3 (malt: water) in a stainless-steel mash tun preheated to 45 °C. The mixture was stirred continuously for 10 min to ensure homogeneity. The mash was heated to 52 °C and held for 20 min to promote protein degradation. The temperature was raised to 63 °C (1 °C/min) and maintained for 40 min to optimize β-amylase activity for maltose production. It was further heated to 72 °C (1 °C/min) and held for 30 min to allow α-amylase-mediated starch liquefaction. The temperature was increased to 78 °C for 10 min to terminate enzymatic activity The mash was transferred to a lauter tun, and the wort was separated from the spent grains via filtration. The wort was collected and sparged with sterilized water to achieve the target pre-boil volume. Then, 0.15 U/10 mL of KM-GD was added. After incubation at 30 °C for 2 h, the reaction was terminated in a boiling water bath for 10 min.

After treatment, the wort was cooled to 11 °C, oxygen was introduced to an oxygen concentration of 9 ppm, and *Saccharomyces cerevisiae* was added to start the fermentation; we maintained a pressure of 0.1 MPa, sealed the fermentation when the sugar level was reduced to 4 for 10 days, and then lowered the temperature to 0 °C to remove the yeast precipitation and obtain a matured beer. The reaction mixture components were detected using HPLC. After filtration (0.22 μm; Millipore, Boston, MA, USA), 10 μL of the sample was injected inside a ZORBAX SB-Aq C_18_ column (L.N. B15072, Agilent, Santa Clara, CA, USA), and the UV absorption spectra were recorded at 254 nm. HPLC analyses were conducted at 40 °C with a 1 mL/min flow rate on a Shimadzu HPLC system equipped with a 2998 photoelectric diode array detector. The mobile phase was 0.02 mol/L potassium dihydrogen phosphate, adjusted to pH 2.8 using phosphoric acid. Guanine standards were obtained from Sigma, St. Louis, MO, USA [16].

## 3. Results

### 3.1. Cloning and Analysis of Guanine Deaminase Genes

The nucleotide sequences of the four guanine deaminases were retrieved from the NCBI database and codon-optimized for transcription and translation in *E. coli* BL21 (DE3). As shown in Table 1, the guanine deaminase genes were synthesized and ligated into pCDFDuet-1 expression vectors. The guanine deaminase genes were 1491 bp, 1503 bp, 1341 bp, and 1317 bp in length. Moreover, their predicted molecular weights were 55.87 kDa, 56.72 kDa, 50.59 kDa, and 49.29 kDa. The sequence similarity of guanine deaminase between *Kluyveromyces lactis* and *Kluyveromyces marxianus* was high (a percentage identity of 68.60% and a query cover of 99%), and the sequence similarity of guanine deaminase between *Lentilactobacillus kefiri* and *Lactobacillus buchneri* was equally high (a percentage identity of 80.41% and a query cover of 97%). However, the sequence similarity of guanine deaminase between *Kluyveromyces lactis* and *Lentilactobacillus kefiri* was much lower (a percentage identity of 33.09 and a query cover of 79%).

**Figure 1:**
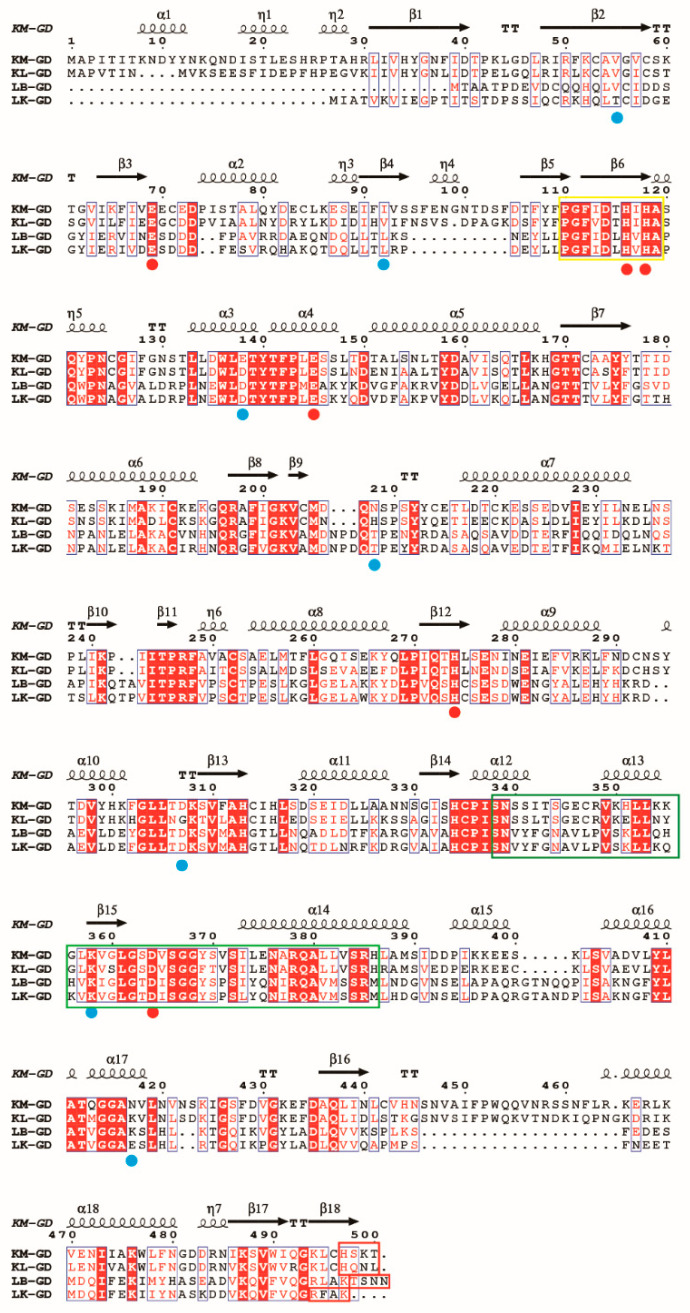
Multiple sequence alignment of guanine deaminase from various sources. KM-GD: amino acid sequence of GDA from Kluyveromyces marxianus; KL-GD: amino acid sequence of GDA from Kluyveromyces lactis; LB-GD: amino acid sequence of GDA from Lactobacillus buchneri; LK-GD: amino acid sequence of GDA from Lentilactobacillus kefiri. Around 106 conserved amino acid sequences are labeled using red backgrounds, conserved substitutions are labeled with blue boxes, residues binding to the substrate and forming hydrogen bonds are marked with blue-filled circles, and amino acid residues within the active site are depicted using red-filled circles. The Zn^2+^-binding domain (yellow square) is well conserved among the four species, while the CRMP homology (green squares) and the PDZ-binding domains (red squares) are less conserved. The figure was built using ESPript through the online ESPript3 server (http://espript.ibcp.fr/ESPript/ESPript/, accessed on 11 December 2024).

Multiple sequence alignment was performed using ESPript. Conserved residues are necessary for deciphering the structural and functional aspects of guanine deaminase. As shown in Figure 1, all guanine deaminases share 106 completely conserved active site residues and account for 20% of the total amino acids. The sequence of guanine deaminase is more conserved than that of previously studied uricases [16]. The guanine deaminase family contains one conserved Zn^2+^-binding domain, one tubulin-binding collapsin response mediator protein (CRMP) homology domain, and one PDZ-binding domain [9]. These conserved amino acids characterize the guanine deaminase active sites. As shown in Figure 1, the Zn^2+^-binding domain is marked in a yellow square, and it is highly conserved (70%). The CRMP homology domain and PDZ-binding domain are only partially conserved. This is consistent with the findings of previous studies [3,9].

### 3.2. Expression of Guanine Deaminase in E. coli

**Figure 2:**
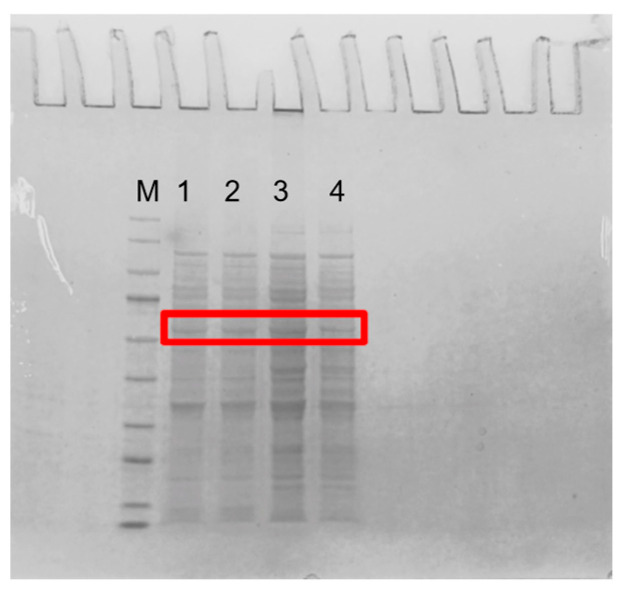
SDS-PAGE of guanine deaminase expressed in *E. coli*. M: marker (9–170 kDa); 1: KM-GD; 2: KL-GD; 3: LB-GD; 4: LK-GD. The guanine deaminases are labeled with red box.

Four guanine deaminase genes were overexpressed in *E. coli* BL21 (DE3) under the T7 promoter in plasmid pCDFDuet-1. Guanine deaminases have been reported to have molecular masses per subunit of between 50 and 60 kDa [28,29,30]. Figure 2 shows that the molecular weights of KL-GD and KM-GD were ~55 kDa, smaller than the previously highlighted molecular weight of 59 kDa for guanine deaminase obtained from human liver [31]. The molecular weights of LB-GD and LK-GD were ~50 kDa, similar to the previously described *Escherichia coli* GDA [32] and smaller than most reported GDAs, such as those from *Arxula adeninivorans* LS3, tea leaves, and rabbit liver [4,14,33].

**Figure 3:**
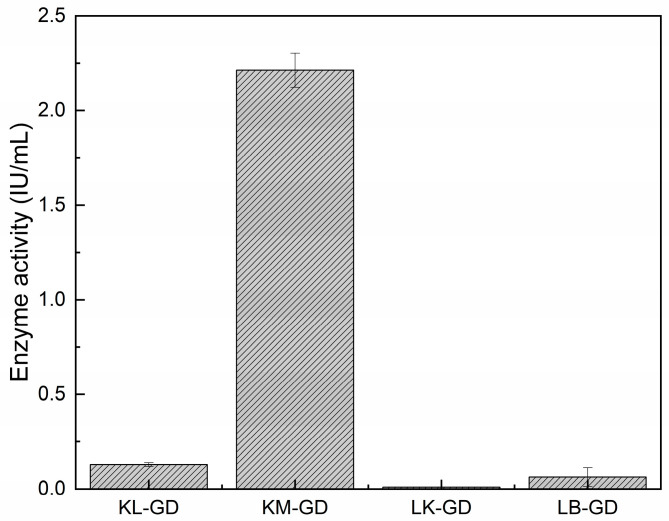
Enzyme activities. All the assays were implemented in triplicate and are represented by mean ± standard deviations.

Crude enzyme activity was tested at 37 °C and pH 7.0. Figure 3 shows that KM-GD (2.21 IU/mL), KL-GD (0.13 IU/mL), and LB-GD (0.06 IU/mL) could be expressed in *E. coli* and catalyze guanine degradation. KM-GD showed the greatest guanine degradation activity. The guanine deaminases of *Escherichia coli* and *Kluyveromyces lactis* were also cloned and overexpressed in *Escherichia coli*, but the enzyme activity was not mentioned. Arxula adeninivorans recombinant guanine deaminase (Agdap) expressed in *Escherichia coli* exhibited an enzyme activity of 0.32–0.43 U/mL. The enzyme activity of KM-GD is 7 times higher than that of Agdap [15,34]. Therefore, KM-GD was chosen for subsequent experiments and characterization.

### 3.3. Enzymatic Properties of KM-GD

**Figure 4:**
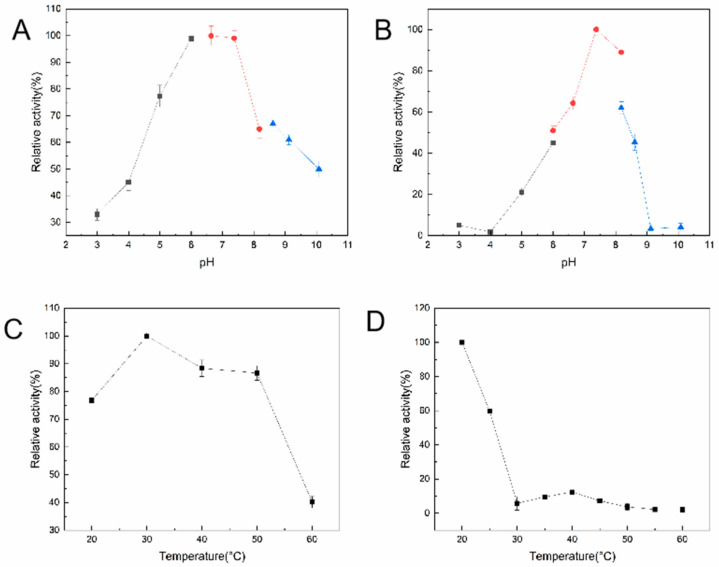
Temperature and pH effects on activity and stability of KM-GD. (**A**) Activity–pH profile; (**B**) stability–pH profile; The sodium acetate (pH 3.0–5.0) is black line, sodium phosphate (pH 5.5–8.0) is red line and Tris-HC1 (pH 8.5–10.0) is blue line; (**C**) activity–temperature profile; (**D**) stability–temperature profile.

Figure 4 shows the effects of temperature and pH on the enzyme activity and stability of KM-GD. As shown in Figure 4A, the catalytic pH of KM-GD was analyzed in a range from pH 2 to pH 10, and the greatest enzyme activity was demonstrated at pH 6.5 in 50 mM Tris-HCl buffer. KM-GD showed high stability between pH 6.5 and 8.0, with residual enzyme activity above 60% (Figure 4B). The catalytic temperature of KM-GD ranged from 25 °C to 50 °C, with an optimal temperature of 30 °C (Figure 4C). With the increase in temperature, the relative enzyme activity first increased and then decreased. The enzyme activity of KM-GD remained above 60% at 25 °C and 30 min (Figure 4D). With the increase in temperature, the relative enzyme activity gradually declined.

The optimal pH of KM-GD was 6.5, which differs from that of the GDAs from lingcod muscle (pH 6.0) [35]; is lower than that of the GDAs from tea leaves (pH 8.5) [33], human liver (pH 8.0) [31], and *Kluyveromyces lactis* (pH 9.0) [15]; and is the same as that of the GDAs from *Arxula adeninivorans* LS3 and recombinant *Arxula adeninivorans* LS3 (pH 6.5) [14]. The optimal reaction temperature was consistent with that of previously reported GDAs from *Kluyveromyces lactis* [15]; however, it was lower than that of the GDAs from *Arxula adeninivorans* LS3 (55 °C), recombinant *Arxula adeninivorans* LS3 (55 °C) [14], and tea leaves (40 °C) [33]. The above GDAs were assayed under their optimum conditions. In addition, all GDAs showed moderate thermostability. The GDA from *Kluyveromyces lactis* retained >50% activity at 40 °C [15]. Furthermore, the thermostability of recombinant guanine deaminase was found to be greatly affected. The optimum temperature was similar to that of endogenous and recombinant GDAs from *Arxula adeninivorans* LS3 (55 °C); however, after incubation for 1 h, the thermostability was higher for endogenous Agdap (60 °C) than for recombinant Agda6hp (30 °C) [14].

### 3.4. Computational Analysis for the Mechanism Underlying Guanine Deaminase Catalysis

**Figure 5:**
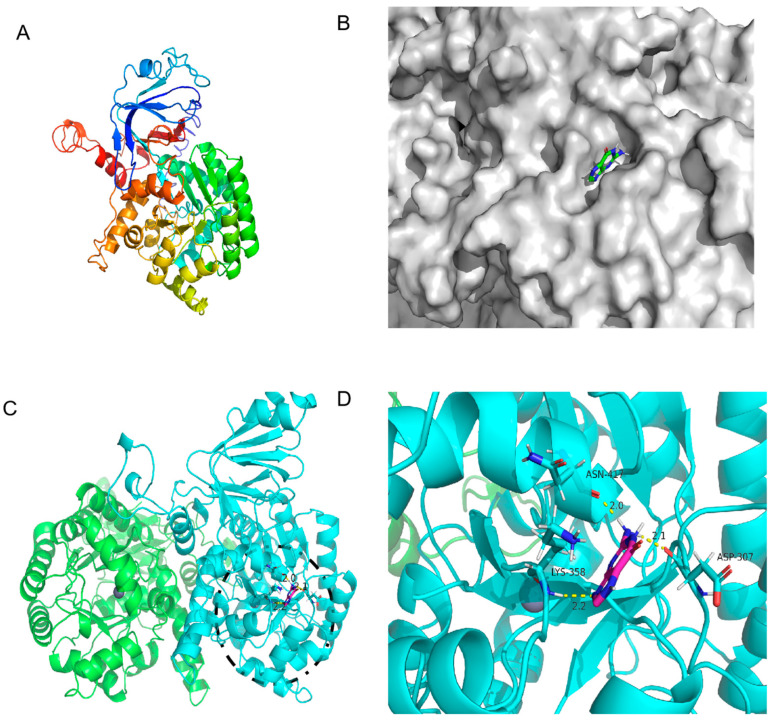
The 3D structure of KM-GD guanine deaminase homology modeling and monomer structure. (**A**) Monomer structure of KM-GD; (**B**) protein surface model of KM-GD and guanine; (**C**) structures of KM-GD oligomers (subunits A and B are marked in different colors), the binding of enzyme and substrate is marked by black circles and shown enlarged in the Figure 5D; (**D**) hydrogen bonding between KM-GD and guanine.

The amino acid sequence of GDA and the properties of the enzyme, particularly the effect of the critical active site on enzymatic properties, were analyzed to explain the property differences among the three GDAs. The structure of the KL-GD monomer was built with the I-TASSER server using yeast guanine deaminase (PDB ID: 6OH9) as the primary template, as shown in Figure 5A. The estimated TM-score was 0.73 ± 0.11, and the estimated RMSD was 7.0 ± 4.1. The X-ray structure of the experimentally solubilized guanine deaminase from yeast (SMTL ID:6oh9.1. A) was used as a template to build the theoretical structure, which shared a 51.88% sequence identity and a homology modeling GMQE score of 0.82 with KL-GD. The homology model was validated using SWISS-MODEL’s QMEANDisCo global score (0.82 ± 0.05). The X-ray structure of the experimentally solubilized guanine deaminase from yeast (SMTL ID:6oh9.1. A) was also used as a template to build the three-dimensional structure of KM-GD. Therefore, it shared a 51.63% sequence identity with KM-GD and a homology modeling GMQE score of 0.80. The homology model was validated using SWISS-MODEL’s QMEANDisCo global score (0.82 ± 0.05). A homodimer three-dimensional structure of LB-GD was developed using the human guanine deaminase X-ray structure as a template (SMTL ID: 2zu9.1. B). The sequence identity of LB-GD was 37.86%, and the homology modeling GMQE score was 0.71. The homology model was validated using SWISS-MODEL’s QMEANDisCo global score (0.69 ± 0.05). The overall structures of the three homology models were similar, with a high confidence level.

As shown in Figure 5C, KM-GD is a homodimer that comprises two subunits, each with a molecular mass of 55 kDa; it also has a canonical (β/α)_8_ TIM barrel fold, which is common to all AHS enzymes, and it contains a single zinc cation with a trigonal bipyramidal coordination geometry [36]. The molecular docking of the protein 3D structure model constructed using homology modeling was performed with the guanine molecule in order to analyze the property differences. The docking results of KM-GD are shown in Figure 5D. The surface model in Figure 5B indicates that the guanine molecule is in an active pocket of KM-GD, facilitating substrate entry and exit. This demonstrates the excellent catalytic ability of KM-GD. The residues involved in the active pocket include ALA198, PHE199, PRO238, LEU239, ILE240, LYS241, LEU269, PRO270, ASP307, LYS308, SER309, ALA326, ASN329, SER330, GLY331, GLY356, LEU357, LYS358, VAL418, LEU419, ASN420, and VAL421. Several highly conserved amino residuals of KM-GD, such as ASP307, are consistent with previous results (ASP327 of *Escherichia coli* and *Saccharomyces cerevisiae* GDA [36]). According to the results of autodock4, the lowest binding energy of KM-GD and guanine is −3.44 kcal/mol. Three residues of KM-GD—ASP307, LYS358, and ASN417—developed a total of three hydrogen bonds with the guanine molecule. ASP307 and the guanine molecule formed 2.1 Å. LYS358 and the guanine molecule formed a hydrogen bond of 2.2 Å. ASN417 and the guanine molecule formed a hydrogen bond of 2.0 Å. ASP307 is a conserved substitution, LYS358 is a conserved animo residual, and ASN417 is a non-conservative residue. The active and hydrogen bond binding sites are distant in the primary structure. Substituting residues outside the conserved region can preserve the structural and functional characteristics of the enzyme.

**Figure 6:**
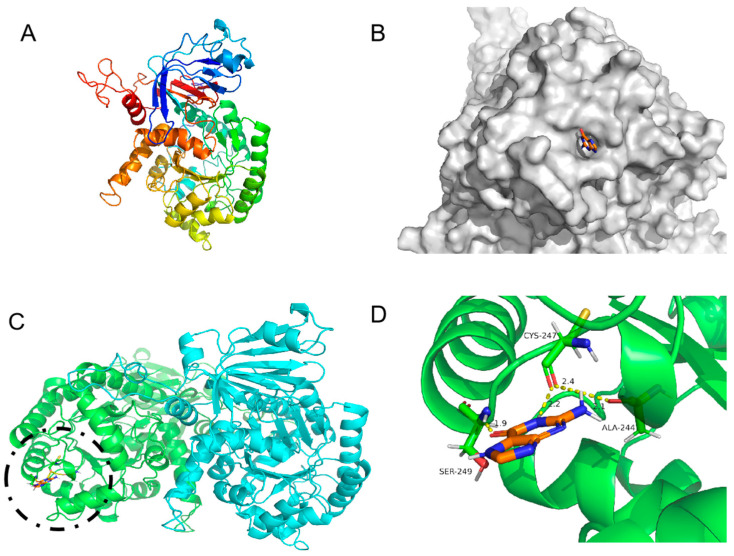
Three-dimensional structure of KL-GD guanine deaminase homology modeling and monomer structure. (**A**) Monomer structure of KL-GD; (**B**) protein surface model of KL-GD and guanine; (**C**) structures of KL-GD oligomers (subunits A and B are marked in different colors), the binding of enzyme and substrate is marked by black circles and shown enlarged in the Figure 6D; (**D**) hydrogen bonding between KL-GD and guanine.

**Figure 7:**
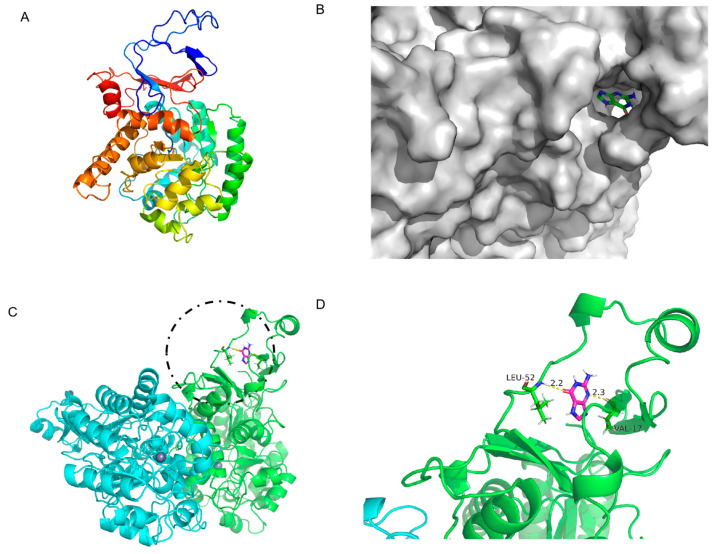
Three-dimensional structure of LB-GD guanine deaminase homology modeling and monomer structure. (**A**) Monomer structure of LB-GD; (**B**) protein surface model of LB-GD and guanine; (**C**) structures of LB-GD oligomers (subunits A and B are marked in different colors), the binding of enzyme and substrate is marked by black circles and shown enlarged in the Figure 7D; (**D**) hydrogen bonding between LB-GD and guanine.

Although KL-GD, KM-GD, and LB-GD had similar structures, their enzyme activities significantly differed. Molecular docking was performed for KL-GD and LB-GD (Figure 6 and Figure 7). The residues of KL-GD involved in the active pocket included HIS111, LEU129, HIS203, SER204, AARG242, PHE243, GLN267, THR268, HIS 269, VAL305, LEU306, ALA307, and GLY362. According to the result of autodock4, the lowest binding energy of KL-GD and guanine is −3.62 kcal/mol and the lowest binding energy of LB-GD and guanine is −3.37 kcal/mol. KL-GD formed three hydrogen bonds with guanine. ASP133 and the guanine molecule developed two hydrogen bonds at 1.8 Å and 3.5 Å, respectively, and HIS203 formed a 2.1 Å hydrogen bond with guanine. The residues of LB-GD involved in the active pocket included THR2, PRO6, ASP7, GLU8, VAL9, ASP10, GLN12, GLN13, HIS14, GLN15, LEU16, VAL17, LEU49, LEU50, THR51, LEU52, SER54, THR57, LEU58, LEU59, GLN383, and ILE384. LB-GD and guanine formed two hydrogen bonds. VAL17 and guanine formed one hydrogen bond at a distance of 2.3 Å, and LEU52 and guanine formed a hydrogen bond of 2.2 Å. KL-GD had a higher substrate-binding capacity than LB-GD. The protein surface model in Figure 6B and Figure 7B revealed that the active pocket of KL-GD was shallow compared with that of KM-GD, and LB-GD had a tunnel-like active pocket compared with that of KM-GD and KL-GD, which is not conducive for guanine substrate entry and could prevent catalysis. The GDAs from *Lactobacillus* and yeast significantly differed; the active pocket location of LB-GD differed from that of two guanine deaminases from yeast.

The structural analysis revealed three critical residues (ASP307, LYS358, and ASN417) in KM-GD that collectively mediate hydrogen bonding interactions with the guanine substrate, forming a total of three stabilizing contacts. Notably, comparative studies with homologous GDAs (e.g., KL-GD and LB-GD) demonstrated that KM-GD exhibits enhanced substrate recognition capabilities through expanded residue participation (involvement of three key catalytic residues) and an optimized hydrogen-bond network (formation of three substrate-anchoring bonds). GDAs typically utilize a divalent metal center Zn^2+^ to mediate the hydrolysis of amide or ester bond-containing substrates. Hybrid quantum mechanics/molecular mechanics (QM/MM) calculations have shown that the first Zn-bound proton transfer to the N3 atom of the substrate is mediated by the E79 residue, and the second proton is transferred to the amine nitrogen of the substrate via E143 [37]. This suggests that the distance between the substrate and the metal ion can also have an effect on the rate of the reaction, and the docking results of the three molecules show that the N3 of guanine is orientated towards Zn^2+^, which is in accordance with the principle of the guanine deamination reaction reported in previous work. At the same time, it is expected to have superior activity compared with other GDAs.

### 3.5. Application of KM-GD to Reduce Guanine in Beer

Beer contains complex purine substances, of which the total content of guanine and guanine nucleosides accounts for about 60% of the total purine content. By using HPLC, it was found that the total purine content in the saccharified wort with a brix of 12 °P was 53.24 mg/L, and the total purine content in the pasteurized beer after fermentation was 21.61 mg/L, indicating that the free purines and bases in the saccharified wort after yeast fermentation were assimilated by the *Saccharomyces cerevisiae*. The guanine content in the saccharified wort was 23.75 mg/L, accounting for 44.6% of the total purine content, and the guanine content in the beer was 3.85 mg/L, accounting for 20.77% of the total purine content.

**Figure 8:**
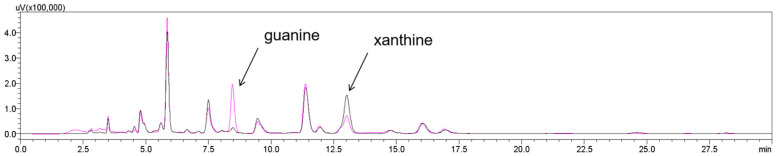
HPLC analysis of guanine of saccharified wort: untreated (black line); treated using KM-GD (purple line). Guanine peak decreased and xanthine peak intensity increased, suggesting the conversion of guanine into xanthine.

Therefore, this study designed a pathway from guanine to hypoxanthine in order to verify the feasibility of using guanine deaminase to degrade guanine in the original wort. Purine degradation and wort saccharification occurred simultaneously. The pH value of the wort after saccharification was 6.04, and the pH value of the saccharified liquor after boiling for 2 h decreased to 5.64; KM-GD had high enzyme activities under both. KM-GD was added to the original wort to degrade guanine. As shown in Figure 8, after the wort saccharification process and KM-GD treatment, the guanine peaks significantly decreased in the saccharified wort, as identified through HPLC. The guanine content of the saccharified wort exhibited a significant decrease from 2.23 mmol to 0.44 mmol, representing an 80.34% reduction in concentration. The guanine content of matured beer exhibited an 80.00% reduction in concentration. Conventional purine-reducing strategies generally use adsorption based on porous media such as chitosan, artificial zeolite, and activated carbon, and the purine removal rates were 84.8%, 65%, and 65%, respectively [38,39]. But the adsorption method is non-specific, and it is difficult to avoid effects on other nutrients or flavor substances. KM-GD has a better removal rate and specificity. It provides potential ideas for the subsequent production of low-purine beverages.

Pasteurized beer is a complex system rich in phosphate-buffered pairs; thus, the pH needs to be adjusted for adaptation to the optimum reaction pH of the enzyme. This is uneconomical and difficult to achieve in industrial production, so it is important to identify guanine deaminases with a suitable pH value. Alkaline rUOX was used to reduce uric acid in beer, beef, and yeast extract at pH 10, which is not suitable for beer fermentation systems [27]. Adjusting the pH of pasteurized beer affects its flavor compounds, resulting in less clarity and lower carbon dioxide solubility. We redesigned the beer production process by adding guanine deaminase to remove guanine from the raw material at the saccharification stage, which can avoid the flavor changes caused by adding enzymes to pasteurized beers. KM-GD has been proven to have good enzyme activity and stability in saccharified wort. Inosine, guanosine, and other purine precursors are also present in beer; thus, purine precursors also need to be taken into account in the preparation of low-purine food using enzymatic digestion.

## 4. Conclusions

This study identified and applied a guanine deaminase (KM-GD) derived from *Kluyveromyces marxianus*, which was screened for its exceptional enzymatic activity (2.21 IU/mL) and optimal pH compatibility (pH 6.5–8) with standard beer brewing conditions. Building upon this discovery, we developed an innovative degradation protocol to specifically eliminate guanine from brewing matrices while preserving essential beer components. The implementation of this strategy achieved remarkable reductions in guanine content, with an 80.34% decrease observed in wort during saccharification and an 80.00% reduction maintained through fermentation to the final product.

This enzymatic approach demonstrates significant advantages over traditional purine-removal methods by maintaining critical flavor profiles and nutritional integrity through substrate-specific catalysis and establishing a scalable bioprocessing framework for low-purine beverage production.

The methodology not only addresses the growing demand for healthier beer alternatives, but also offers valuable insights for industrial brewing applications to produce low-purine beverages.

## Figures and Tables

**Table 1:** Guanine deaminase genes involved in this study.

Gene	Species	Serial Number	Enzyme Site	Sequence Length (bp)
*kl* *-* *gud*	*Kluyveromyces lactis*	Gene ID: 2893421	*Eco*RI/*Nde*I	1491
*km* *-* *gud1*	*Kluyveromyces marxianus*	Gene ID: 34717204	*Hin*dIII/*Kpn*I	1503
*Lk-guaD*	*Lentilactobacillus kefiri*	Gene ID: 71567331	*Eco*RI/*Kpn*I	1341
*Lb-guaD*	*Lactobacillus buchneri*	Gene ID: 72460628	*Eco*RI/*Kpn*I	1317

## Data Availability

The original contributions presented in this study are included in the article. Further inquiries can be directed to the corresponding author.

## Abbreviations

The following abbreviations are used in this manuscript:

GDA	guanine deaminase
AHS	amidohydrolase superfamily
NCBI	National Center for Biotechnology Information database
KM-GD	guanine deaminase of *Kluyveromyces marxianus*
KL-GD	guanine deaminase of *Kluyveromyces lactis*
LK-GD	guanine deaminase of *Lentilactobacillus kefiri*
LB-GD	guanine deaminase of *Lactobacillus buchneri*
